# A Case of Abscess in the Superior Maxillary Sinus Not Due to a Diseased Tooth

**Published:** 1881-09

**Authors:** 


(*Hubschmann in Deutsche Vierteljahrschrift fur Zahnheilkunde fur July*, 1881.)

### A CASE OF ABSCESS IN THE SUPERIOR MAXILLARY SINUS NOT DUE TO A DISEASED TOOTH.

At the beginning of last November, a woman consulted me in regard to her husband's condition. According to her statement there was marked swelling on the left side of his face, the nasal passage on that side being closed and discharging foetid mucous. This condition had existed for five weeks. His physician had diagnosed it as a simple catarrhal affection, from which he would recover shortly without any treatment. As he was not able to come to me, on account of an affection of the spinal cord, I prescribed a solution of potassium permanganate as a nasal injection, and promised to see him the next day. I found him with a swelling that extended from the canine to the first molar, and reached as high up as the margin of the orbit. The outer wall of the nose was forced in so far that the nasal passage was occluded. An examination revealed a denture that was well developed, and, with the exception of the second molar, free from caries. Pressure or tapping on the teeth failed to develop any tenderness, but as the patient was desirous of having the curious tooth extracted, I removed it, but found no connection between its socket and the antrum. I was obliged to find an outlet for the pus, for it was evident that an abscess existed from the foetid nasal discharge, but owing to his general condition, it was not deemed advisable either to extract one or more teeth, or to resort to any of the trephining operations. I therefore ordered a warm chamomile poultice in order to promote suppuration, supposing that the abscess would point at the root of one of the teeth. A few days afterwards, I found the first bicuspid tender and pus discharging around it whenever the lip was raised. I extracted the tooth and found a small opening in the socket communicating with the autrum, which I enlarged, allowing quite a quantity of pus to discharge. The swelling about the eye was very marked, and at the lower margin of the orbit was hard and of a copper brown color. On the following day, pus was found to exist in the swelling. For injecting into the autrum, I ordered a carbolic acid solution. A few days afterward the swelling had subsided, the nasal passage was open, and the foetid mucus discharge had ceased. The discharge of pus continued for eight days, and was followed by a watery discharge for several days. Since that time he has been well.

(*Hubschmann in Deutsche Vierteljahrschrift fur Zahnheilkunde fur July*, 1881.)

Query by translator. Would it not have been better to have entered the autrum through the socket of the extracted molar, and given exit to the pus, than by the delay to have sacrificed the first biscupid, and allowed an abscess to form under the margin of the orbit?

